# The Effects of Inulin on the Growth, Oxidative Stress, and Immune Function of Weaned Kids

**DOI:** 10.3390/ani15162455

**Published:** 2025-08-21

**Authors:** Zhiling Zhou, Chunmei Du, Pengxin Wu, Jian Ma, Shangquan Gan, Zhijing Wang, Fuquan Yin

**Affiliations:** 1 College of Coastal Agriculture Science, Guangdong Ocean University, Zhanjiang 524088, China; 2 Qingyuan Animal Disease Control Center, Qingyuan 511500, China

**Keywords:** antioxidant ability, inulin, intestinal health, immune indicators, weaned kids

## Abstract

Goats are important producers of meat, milk, hair, and leather. Early-weaned kids may encounter issues such as weaning stress and stress from high-density farming, which can hinder their growth and development. Therefore, exploring feed additives capable of alleviating stress in kids to enhance their growth performance is of particular importance. This experiment aims to investigate the effects of inulin on the growth performance, immune function, and intestinal health of weaned kids. Thirty healthy 60-day-old Leizhou black goat weaned kids with similar body weights (9.00 ± 0.05 kg) were selected and evenly divided into five groups by weight, with six kids in each group. The control group was fed the basal diet, while the AM HCl group received 0.05‰ aureomycin hydrochloride. The inulin groups were supplemented with 0.1%, 0.3%, and 0.5% inulin, respectively. By comparison, the results of the 0.3% inulin group in this experiment were most consistent with those of the AM HCl group, and the final weight of the kids was the highest. The 0.3% and 0.5% inulin groups had significantly enhanced immune-related indicator (such as sIgA, IgA, and IgG) concentrations and antioxidant activities (*p* < 0.05) in kids. Inulin significantly increased cecal total volatile fatty acids (TVFA) and the activities of jejunal α-amylase, chymotrypsin, and trypsin (*p* < 0.05); the activities of intestinal antioxidant enzymes, including GSH-PX, SOD, and T-AOC, were significantly increased (*p* < 0.05); and the intestinal anti-inflammatory factor IL-10 was significantly elevated (*p* < 0.05). Inulin significantly increased the mRNA expression levels of the tight junction protein genes *TJP1 (ZO-1)* and *OCLN* in the jejunum (*p* < 0.05). It also significantly upregulated the mRNA expression levels of anti-inflammatory factors *TGF-β1* and *IL-10* while significantly reducing the mRNA level of pro-inflammatory factor IL-6 in the jejunum (*p* < 0.05). Therefore, inulin enhanced the growth performance and antioxidant capacity of weaned kids, improved the body’s immune response, and reduced inflammatory responses. Specifically, when comparing the growth status, antioxidant enzyme activity, and tight junction protein mRNA level among the groups of kids, the 0.3% inulin supplementation yielded the best results in this experiment.

## Simple Summary

Weaning stress and intensive farming significantly impair growth performance in lambs. Amidst global restrictions on antibiotic use, inulin has emerged as a viable alternative to growth-promoting antibiotics, offering multiple beneficial properties. As a prebiotic, it enhances nutrient absorption capacity, exhibits anti-inflammatory activity, and mitigates physiological stress responses in livestock. This study examined the effects of dietary inulin supplementation in weaned kids. The findings indicate that a 0.3% inulin inclusion rate optimally enhances growth parameters, reduces inflammatory markers, and upregulates intestinal tight junction protein expression.

## 1. Introduction

Leizhou goats are characterized by a short breeding cycle, high fecundity, strong adaptability, and fine meat quality. Consequently, in the Leizhou area of Zhanjiang, there are as many as 300 professional households engaged in Leizhou goat farming. Weaned kids were selected as the experimental subjects because intensive goat farming is a profitable venture in China, and kids reared under such conditions are particularly prone to illness due to stress. Young livestock exhibit high susceptibility to stressors, which significantly elevate cortisol secretion, thereby disrupting the homeostasis of the internal environment and impairing metabolism [1]. Concurrently, stressors impair intestinal digestive function, leading to stunted growth and emotional instability [2]. Weaning stress response not only impairs kids’ health and growth but also elevates their risk of disease, resulting in economic losses for goat farming. In young animals, the immune system is underdeveloped, and the weaning process often induces oxidative stress [3], leading to an imbalance in the intestinal microbiota and subsequent problems such as infections [4]. Antioxidant compounds derived from feed sources can enhance the intestine’s antioxidant capacity, thereby mitigating the adverse effects of oxidative stress on the intestine [5].

In the contemporary global food consumption landscape, animal products have emerged as a pivotal component in addressing consumers’ nutritional needs and improving dietary profiles. Nonetheless, amidst growing public scrutiny over feed safety and animal welfare, the application of antibiotic growth promoters (AGPs) in the production of meat animals has come under increasing scrutiny and regulatory restrictions [6]. This trend has prompted researchers and practitioners to actively explore safe alternatives to AGPs, aiming to mitigate food safety concerns and meet the market’s demand for green and healthy animal products. The excessive use of antibiotics in animal husbandry may precipitate the emergence of bacterial resistance [7]. Research has demonstrated that antibiotics can induce bacteria within biofilms to expel antibiotics by overexpressing efflux pumps, thereby contributing to antibiotic resistance [8]. Additionally, Gram-negative bacteria within biofilms utilize the quorum sensing (QS) system to facilitate the secretion of virulence factors, thereby exerting adverse effects on the body [9]. As a result, the development of natural extracts containing the fructan inulin to interfere with QS activity and biofilm formation can be regarded as a novel approach. Inulin is a major type of fructan, with chicory roots, Jerusalem artichoke tubers, and chicory flower buds serving as its primary botanical sources [10]. Inulin exhibits good stability under acidic and high-temperature conditions and can maintain its activity for an extended period [11]. It activates nuclear factor erythroid 2-related factor (Nrf2) and Kelch-like ECH-associated protein 1 (Keap1), thereby potentiating the activity of intracellular antioxidant enzymes [12]. Additionally, as a prebiotic, it provides health benefits, such as reducing blood sugar and cholesterol concentrations [13]. Inulin is a prebiotic and belongs to a type of fructooligosaccharide, and the unique structure prevents it from being digested by animal digestive enzymes, allowing it to act directly on the intestines and thereby increase the activities of intestinal antioxidant factors [14]. Herosimczyk A et al. [15] found that inulin supplementation could increase the selenium content in the kidneys and liver for enhancing the antioxidant capacity of piglets. Jonova S et al. [16] demonstrated that inulin promoted the development of the rumen and small intestines in calves and exerted a positive effect on their digestive and absorptive functions. Suthama N et al. [17] found that the addition of inulin increased the immunoglobulin concentrations in young chickens and enhanced their ability to resist pathogen invasion.

This study aimed to evaluate the effects of inulin on growth performance, serum biochemical parameters, antioxidant indices, immune indices, the intestinal physical barrier, and the chemical intestinal barrier in weaned Leizhou black goats. The results of this experiment provide a reference for the application of inulin as an antioxidant regulator in weaned Leizhou black goats and as a potential antibiotic substitute in livestock production using plant fructans.

## 2. Materials and Methods

### 2.1. Kids and Experimental Protocol

In this experiment, inulin with a purity of 99% was purchased from Fuyang Dianpeng Food Additive Co., Ltd. (Fuyang, China), and the original powder of the livestock and poultry antibiotic aureomycin hydrochloride (AM HCl) was purchased from Huizhou Hongtai Biotechnology Co., Ltd. (Huizhou, China).

Thirty 60-day-old Leizhou black goat kids with similar body weights were selected for forced weaning. They were then evenly divided into five groups (n = 6) based on the kids’ body weights, ensuring that the mean weight of each group was similar. The control group was fed the basal diet, while the AM HCl group was supplemented with 5 mg of AM HCl per kilogram of the basal diet. For the 0.1%, 0.3%, and 0.5% inulin groups, 1 g, 3 g, and 5 g of inulin, respectively, were added per kilogram of the basal diet [18,19]. The experimental period lasted 40 days.

The basal diet formulation (Table 1) was designed in accordance with China’s Feed Standard for Meat-Producing Sheep and Goats (NY/T 816-2004). The goat sheds were first cleaned and disinfected. During the experiment, the goats, which were individually penned, were fed once at 08:00 and once at 17:00 daily. Each group of goats had free access to feed, water, and exercise. The amount of feed provided was adjusted daily based on the residual feed of each goat.

### 2.2. Growth Performance

The goats were weighed individually before the morning feeding on the 1st, 26th, and 41st days of the experiment, and their initial body weight (IBW) and final body weight (FBW) were recorded. During the experiment, the daily feed intake of each goat was calculated by systematically monitoring and meticulously recording data on the daily feed supply and residual amounts for the kids. Through statistical analysis, the average daily gain (ADG) and average daily feed intake (ADFI) of the experimental goats were determined. Following the experiment, the feed-to-gain ratio (F/G) of each goat was calculated as follows:

Average daily gain (ADG), g/d = (FBM-IBM)/days of experiment;

Average daily feed intake (ADFI), g/d = (daily feed supply-residual feed)/(days of experiment × the number of goats in each group);

Feed to gain ratio (F/G), % = ADFI/ADG.

### 2.3. Sample Collection

On the 41st day of the trial, blood samples were collected from the jugular veins of the experimental kids. The collected blood was allowed to stand for half an hour. The centrifugation speed was set at 4276× *g*. After 15 min of centrifugation at 4 °C, the serum was separated, transferred to numbered EP tubes, and stored at −20 °C in a refrigerator for subsequent detection of serum indicators. The kids were stunned first before being slaughtered; the kids’ abdominal cavities were then quickly opened; and the duodenum, jejunum, and ileum were separated. Approximately 2 cm segments were cut from the middle part of each small intestine, placed in enzyme-free centrifuge tubes containing 4% paraformaldehyde solution, and used for preparing paraffin sections of each small intestinal segment for subsequent detection of intestinal morphology. The remaining portions of the duodenum, jejunum, and ileum were rinsed with physiological saline, cut into several 2 cm intestinal segments, placed in enzyme-free centrifuge tubes, and stored at −80 °C for subsequent detection of intestine-related indicators. The cecum of the kids was isolated, and its contents were collected using sterile plastic tubes and then stored at −20 °C for further analysis of volatile fatty acids.

### 2.4. Intestinal Histomorphology

To examine the morphology of the intestinal tissues, the ileum, jejunum, and duodenum were removed from the 4% paraformaldehyde solution, followed by dehydration, paraffin embedding, and sectioning. Subsequently, the sections were subjected to H&E staining following the procedures of dewaxing, hydration, hematoxylin staining, eosin restaining, dehydration, and mounting [20]. After the above processing steps were completed, the intestinal microstructures were imaged using the Panoramic MIDI scanner (3D HISTECH, Budapest, Hungary), and the images were analyzed. The intestinal villus height (VH), crypt depth (CD), and villus–crypt ratio (VCR) of the intestinal sections were measured using Case Viewer 2.4 software.

### 2.5. Determination of Serum Biochemical, Antioxidant, and Immune Indexes

Serum biochemical indices, including total protein (TP; Catalog No. A045-4-1), triglyceride (TG; Catalog No. A110-1-1), total cholesterol (TC; Catalog No. A111-1-1), alkaline phosphatase (AKP; Catalog No. A059-1-1), urea nitrogen (UN; Catalog No. C013-2-1), and blood glucose (GLU; Catalog No. A154-1-1), were determined strictly by the instructions of the colorimetry kits (Nanjing Jiancheng Biotechnology Research Institute, Nanjing, China).

The indices of the antioxidant system, such as catalase (CAT; Catalog No. A007-1-1), glutathione peroxidase (GSH-Px; Catalog No. A005-1-2), malondialdehyde (MDA; Catalog No. A003-1), superoxide dismutase (SOD; Catalog No. A001-3), and total antioxidant capacity (T-AOC; Catalog No. A015-1-1), were determined strictly by the instructions of the colorimetry kits (Nanjing Jiancheng Biotechnology Research Institute, Nanjing, China).

The concentrations of immunoglobulins (IgA, Catalog No. MM-28374; IgM, Catalog No. MM-28377; IgG, Catalog No. MM-3525002), cortisol(COR, Catalog No. MM-11062), globulin (GLB, Catalog No. MM-17747), and cytokines (IL-1β, Catalog No. MM-175101; IL-6, Catalog No. MM-35226O1; IL-10, Catalog No. MM-009801; TNF-α, Catalog No. MM-009601; IFN-γ, Catalog No. MM-009501) in the serum were determined using serum immune index kits (Jiangsu Meimian Industry Co., Ltd., Yancheng, China). The determination steps of immune indexes were strictly carried out in accordance with the instructions of the ELISA kits.

### 2.6. The Effects of Intestinal Permeability, Jejunal Digestive Enzymes, and Colonic Volatile Fatty Acids

The prepared serum was used to determine the intestinal permeability-related indices: D-lactic acid (D-LA; Catalog No. G0827W), diamine oxidase (DAO; Catalog No. G0154W), and lipopolysaccharide (LPS; Catalog No. G0902W). The experimental steps were carried out according to the instructions of the ELISA kits (Suzhou Grace Biotechnology Co., Ltd., Suzhou, China).

The detection steps for jejunal digestive enzymes were as follows. The intestinal mucosa of the jejunum was carefully scraped on an ice pack to determine the activities of α-amylase (Catalog No. G0510W), chymotrypsin (Catalog No. G1211W), trypsin (Catalog No. G1209W), and lipase (Catalog No. G0338W). The ELISA kits were used for determining intestinal digestive enzyme indices (Suzhou Grace Biotechnology Co., Ltd., Suzhou, China). The experimental procedures were strictly conducted in accordance with the reagent instructions.

The volatile fatty acids (VFAs) in the cecal contents were detected using the gas chromatography and mass spectrometry conditions described by Kareem et al. [21]. Specifically, 1 g of cecal contents was mixed with 1 mL of 24% sodium metaphosphate solution, left overnight at room temperature, and then centrifuged for 20 min (4 °C, 9977× *g*). The supernatant was supplemented with a 20 mM 4-methylvaleric acid internal standard to a final concentration of 10 mM. The contents of acetic acid, propionic acid, butyric acid, and TVFAs in the cecal samples were analyzed using a gas chromatograph (TRACETM 1310, Thermo Fisher Scientific, Waltham, MA, USA).

### 2.7. Intestinal Antioxidant Index and Immune Performance

To determine the intestinal antioxidant indices and intestinal immune performance indices of the kids, 10% tissue homogenates were prepared from the jejunum, ileum, and duodenum segments according to the instructions of the ELISA kits (Nanjing Jiancheng Bioengineering Institute, Nanjing, China and Jiangsu Meimian Industry Co., Ltd., Yancheng, China). After centrifugation at 4276× *g* for 15 min at 4 °C, the supernatants were collected to measure antioxidant indices, including CAT, GSH-Px, MDA, SOD, and T-AOC, and immune performance indices, including sIgA (Catalog No. MM-75118O1), IgA, IgG, IgM, IL-10, IL-6, IFN-γ, and TNF-α.

### 2.8. qPCR Analysis

RNA was extracted from the scraped jejunal mucosal tissue. Jejunal RNA was extracted using RNAiso Plus (Catalog No. 9108Q, Takara, Tokyo, Japan), and the purity and level of the RNA were then determined using a spectrophotometer (Micro Drop SE, Shanghai BIO-DL Science Instrument, Shanghai, China) for subsequent experiments. The synthesis of the first strand of cDNA was carried out according to the instructions of the Hifair^®^ II 1st Strand cDNA Synthesis Kit (Catalog No. 11119ES60, Vazyme Biotech Co., Ltd., Nanjing, China). Primers were designed based on the mRNA sequences of goat target genes (*TJP1*, *CLDN-3*, *OCLN*, *TGFB-1*, *IL-6*, and *IL-10*) and the internal reference gene *GAPDH* retrieved from the NCBI database. The primers (Table 2) were synthesized by Sangon Biotech Co., Ltd. (Shanghai, China). According to the instruction manual of Hieff^®^ qPCR SYBR Green Master Mix (Low Rox Plus) (Catalog No. 11202ES08, Vazyme Biotech Co., Ltd., Nanjing, China), the relative mRNA expression levels were determined by real-time PCR (qPCR) detecting system (ViiA 7, ABI). Relative mRNA expression levels of *TJP1*, *CLDN3*, *OCLN*, *TGFB1*, *IL-6*, and *IL-10* were calculated using the 2^−∆∆Ct^ method, with *GAPDH* as the normalization control.

### 2.9. Statistical Analysis

The experimental data were initially compiled and organized using Excel 2016. A one-way analysis of variance (ANOVA) was performed with SPSS Statistics 27.0 for data analysis. Passed homogeneity of variance test and Duncan’s multiple range test were employed to identify significant differences among treatment groups; others were identified by Dunnett’s C. Bar charts were created using GraphPad Prism 8. All data were presented as mean ± standard error of the mean (SEM), and statistical significance was set at *p* < 0.05.

## 3. Results

### 3.1. Effects of Inulin on Growth Performance of Weaned Kids GraphPad Prism

As shown in Table 3, the ADG was significantly increased (*p* < 0.05) in the AM HCl, 0.1% inulin, 0.3% inulin, and 0.5% inulin groups by 21.74%, 3.95%, 20.09%, and 13.65%, respectively, compared to the control group. The F/G was significantly reduced (*p* < 0.05) in the AM HCl, 0.3% inulin, and 0.5% inulin groups by 22.04%, 15.53%, and 13.36%, respectively. No significant differences (*p* > 0.05) were observed in ADG during the early stage or ADFI during the late stage among all inulin-supplemented groups.

### 3.2. Effect of Inulin on Intestinal Histomorphology of Weaned Kids

The effects of inulin supplementation on intestinal morphology in weaned kids are presented in Table 4. In the duodenum and ileum, the VH in the inulin groups was significantly higher than that in the control group (*p* < 0.05). The VCR in the duodenum was significantly increased (*p* < 0.05). No significant differences (*p* > 0.05) were observed in the duodenal CD or jejunal VCR between the inulin groups and the control group.

### 3.3. Effects of Inulin on Serum Biochemical Indicators, Antioxidant Indicators, Intestinal Permeability, and Immune Indicators of Weaned Kids

**Figure 1:**
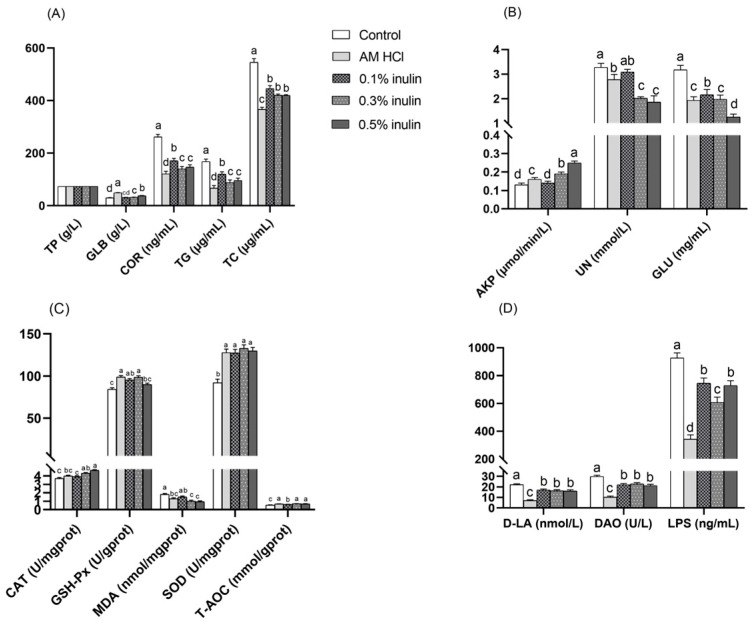
Effects of inulin on serum biochemical indicators (**A**,**B**), serum antioxidant indicators (**C**), and intestinal permeability (**D**) of weaned kids. TP: total protein, GLB: globulin, COR: cortisol, TG: triglyceride, TC: total cholesterol, AKP: alkaline phosphatase, UN: urea nitrogen, GLU: blood glucose, CAT: catalase, GSH-Px: glutathione peroxidase, MDA: malondialdehyde, SOD: superoxide dismutase, T-AOC: total antioxidant capacity, D-LA: D-lactic acid, DAO: diamine oxidase, LPS: lipopolysaccharide. Control: kids were fed with basal diet; AM HCl: kids were fed supplemented with 0.05‰ aureomycin HCl; 0.1% inulin, 0.3% inulin, and 0.5% inulin groups: kids were fed with 0.1%, 0.3%, and 0.5% of inulin, which were added respectively to the basal diet. The results are expressed as the mean ± SEM (n = 6). In the same indicator, different letters indicate significant differences (*p* < 0.05).

According to Figure 1, inulin significantly increased the concentrations of GLB and AKP (*p* < 0.05); conversely, the concentrations of serum COR, TG, TC, UN, and GLU were significantly reduced (*p* < 0.05). Inulin (especially 0.3%) significantly improved antioxidant enzyme activities (CAT, GSH-Px, and SOD) while reducing MDA concentration (*p* < 0.05). Compared with the control group, the contents of D-LA, DAO, and LPS of the inulin group were significantly decreased (*p* < 0.05). There was no significant difference among the inulin addition groups in terms of TP, TC, D-LA, and DAO concentrations as well as SOD activity (*p* > 0.05).

**Figure 2:**
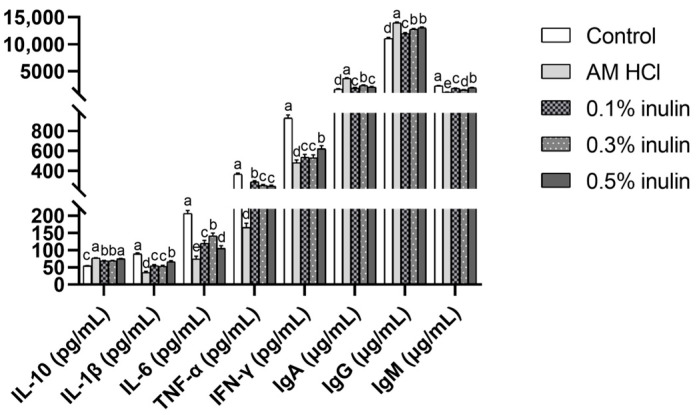
Effects of inulin on serum immune indices of weaned kids. IL-10: interleukin-10, IL-1β: interleukin-1β, IL-6: interleukin-6, TNF-α: tumor necrosis factor-α, IFN-γ: interferon-γ, IgA: immunoglobulin A, IgG: immunoglobulin G, IgM: immunoglobulin M. Control: kids were fed with basal diet; AM HCl: kids were fed supplemented with 0.05‰ aureomycin HCl; 0.1% inulin, 0.3% inulin, and 0.5% inulin groups: kids were fed with 0.1%, 0.3%, and 0.5% of inulin, which were added respectively to the basal diet. The results are expressed as the mean ± SEM (n = 6). In the same indicator, different letters indicate significant differences (*p* < 0.05).

According to Figure 2, inulin significantly increased the concentrations of serum IL-10, IgA, and IgG (*p* < 0.05); conversely, the concentrations of serum IL-1β, IL-6, TNF-α, IFN-γ, and IgM were significantly reduced (*p* < 0.05).

### 3.4. Effects of Inulin on Jejunal Digestive Enzymes and Cecal Volatile Fatty Acids in Weaned Kids

**Figure 3:**
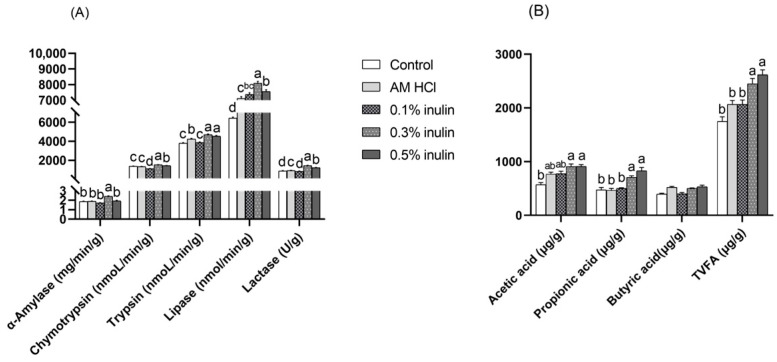
Effects of inulin on jejunal digestive enzymes (**A**) and cecal VFAs (**B**) of weaned kids. TVFAs: total volatile fatty acids. Control: kids were fed with basal diet; AM HCl: kids were fed supplemented with 0.05‰ aureomycin HCl; 0.1% inulin, 0.3% inulin, and 0.5% inulin groups: kids were fed with 0.1%, 0.3%, and 0.5% of inulin, which were added respectively to the basal diet. The results are expressed as the mean ± SEM (n = 6). In the same indicator, different letters indicate significant differences (*p* < 0.05).

As shown in Figure 3, compared with the control group, the activities of jejunal digestive enzymes (chymotrypsin, trypsin, lipase, and lactase) in the inulin groups (0.3% and 0.5%) were significantly increased (*p* < 0.05). Compared with the control group, the concentrations of acetic acid and propionic acid in the cecal contents of the 0.3% inulin and 0.5% inulin groups were significantly increased (*p* < 0.05). There was no significant difference in the contents of butyric acid in the cecum among the groups (*p* > 0.05).

### 3.5. Effects of Inulin Intestinal Antioxidant Performance and Immune Performance of Weaned Kids

**Figure 4:**
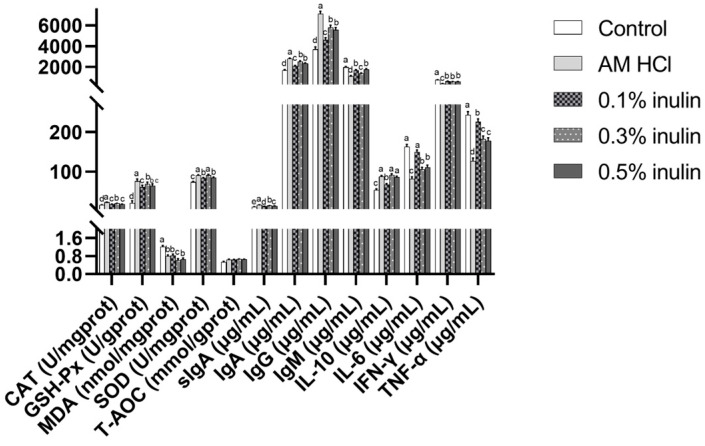
Effects of inulin duodenal antioxidant performance and immune performance of weaned kids. CAT: catalase; GSH-Px: glutathione peroxidase; MDA: malondialdehyde; SOD: superoxide dismutase; T-AOC: total antioxidant capacity; sIgA: secretory immunoglobulin A; IgA: immunoglobulin A; IgG: immunoglobulin G; IgM: immunoglobulin M; IL-10: interleukin-10; IL-6: interleukin-6; IFN-γ: interferon-γ; TNF-α: tumor necrosis factor-α. Control: kids were fed with basal diet; AM HCl: kids were fed supplemented with 0.05‰ aureomycin HCl; 0.1% inulin, 0.3% inulin, and 0.5% inulin groups: kids were fed with 0.1%, 0.3%, and 0.5% of inulin, which were added respectively to the basal diet. The results are expressed as the mean ± SEM (n = 6). In the same indicator, different letters indicate significant differences (*p* < 0.05).

**Figure 5:**
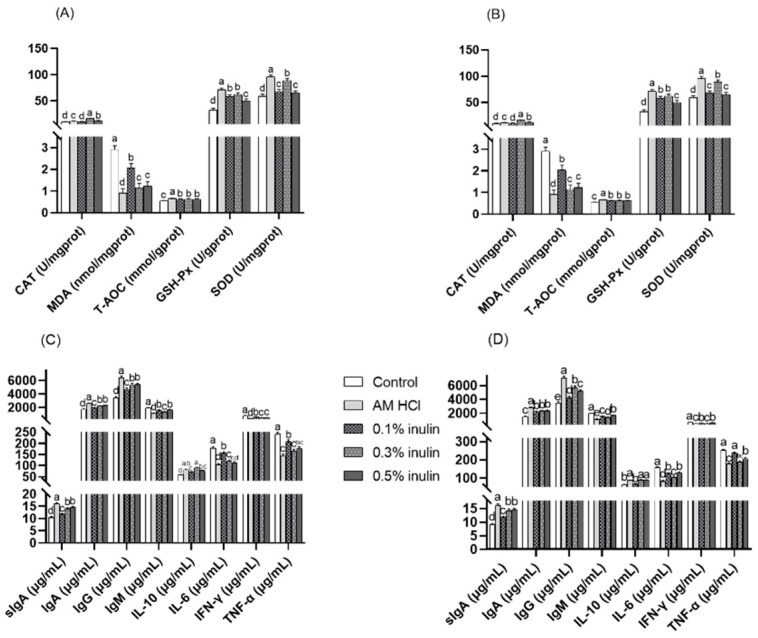
Effects of inulin jejunal antioxidant performance (**A**) and immune performance (**C**), ileal antioxidant performance (**B**), and immune performance (**D**) of weaned kids. CAT: catalase; GSH-Px: glutathione peroxidase; MDA: malondialdehyde; SOD: superoxide dismutase; T-AOC: total antioxidant capacity; sIgA: secretory immunoglobulin A; IgA: immunoglobulin A; IgG: immunoglobulin G; IgM: immunoglobulin M; IL-10: interleukin-10; IL-6: interleukin-6; IFN-γ: interferon-γ; TNF-α: tumor necrosis factor-α. Control: kids were fed with basal diet; AM HCl: kids were fed supplemented with 0.05‰ aureomycin HCl; 0.1% inulin, 0.3% inulin, and 0.5% inulin groups: kids were fed with 0.1%, 0.3%, and 0.5% of inulin, which were added respectively to the basal diet. The results are expressed as the mean ± SEM (n = 6). In the same indicator, different letters indicate significant differences (*p* < 0.05).

As shown in Figure 4 and Figure 5, compared with the control group, the activities of GSH-PX and SOD in each segment of small intestine (duodenum, jejunum, ileum) in the experimental group were significantly increased (*p* < 0.05), while the MDA content was significantly decreased (*p* < 0.05). The CAT activities in the duodenum and ileum of the experimental groups were significantly increased (*p* < 0.05). Additionally, the CAT activities in the jejunum of the AM HCl group, 0.3% inulin group, and 0.5% inulin group were significantly increased (*p* < 0.05). The following showed no significant differences from the control group (*p* > 0.05): in the duodenum, T-AOC in all inulin groups; in the jejunum, CAT in the 0.1% inulin group; and in the ileum, CAT in the 0.1% inulin group.

Compared with the control group, the contents of sIgA, IgA, and IgG in each segment of small intestine (duodenum, jejunum, ileum) in the experimental group were significantly increased (*p* < 0.05), while the contents of IgM and IFN-γ were significantly decreased (*p* < 0.05). The content of IL-10 in the duodenum and jejunum of the experimental group was significantly increased (*p* < 0.05), the content of IL-6 in the jejunum and ileum was significantly decreased (*p* < 0.05), while the content of TNF-α in the duodenum and jejunum was also significantly decreased (*p* < 0.05). The content of IL-6 in the duodenum and the content of TNF-α in the ileum were significantly decreased (*p* < 0.05) in the AM HCl group, 0.3% inulin group, and 0.5% inulin group, while the content of IL-10 in the ileum was significantly increased (*p* < 0.05). In the duodenum, IL-6 in the 0.1% inulin group and, in the ileum, TNF-α in the 0.1% inulin group showed no significant differences from the control group (*p* > 0.05).

### 3.6. Effects of Inulin on the Expression of Intestinal Barrier-Related and Cytokine-Related Genes in the Jejunum

**Figure 6:**
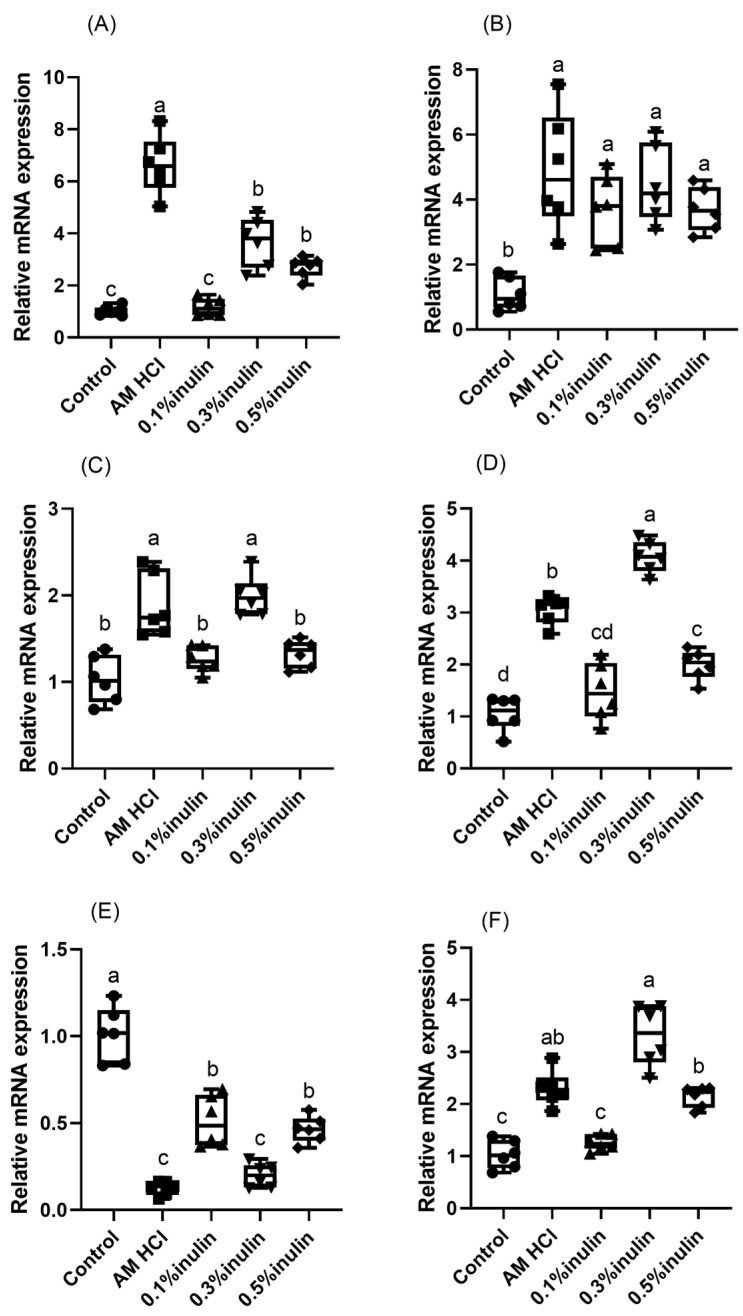
Effects of inulin on expression of *TJP1 (ZO-1)* (**A**), *CLDN-3* (**B**), *OCLN* (**C**)**,** *TGF-β1* (**D**), *IL-6* (**E**), and *IL-10* (**F**) in the jejunum of weaned kids. *TJP1 (ZO-1)*: a key cell junction protein gene that is primarily involved in the formation of tight junctions between cells; *CLDN-3*: a key gene in tight junctions participating in the selective permeability and cell polarization of tight junctions and playing a connecting role in the transmission of substances and energy between cells; *OCLN*: a gene that encodes an integral membrane protein required for cytokine-induced regulation of the paracellular permeability barrier of tight junctions; *TGF-β1*: transforming growth factor-β1, which regulates cell growth, proliferation, differentiation, and apoptosis; *IL-6*: interleukin-6, which is a cytokine associated with inflammation; *IL-10*: interleukin-10, which is a key inhibitory factor in inflammatory responses. Control: kids were fed with basal diet; AM HCl: kids were fed supplemented with 0.05‰ aureomycin HCl; 0.1% inulin, 0.3% inulin, and 0.5% inulin groups: kids were fed with 0.1%, 0.3%, and 0.5% of inulin, which were added respectively to the basal diet. The results are expressed as the mean ± SEM (n = 6). In the same indicator, different letters indicate significant differences (*p* < 0.05).

As shown in Figure 6, compared with the control group, the expression levels of *TJP1 (ZO-1)*, *OCLN*, *TGF-β1*, and *IL-10* in the jejunum of the 0.3% and the 0.5% inulin groups were significantly increased (*p* < 0.05). The expression levels of *CLDN-3* in the jejunum of the experimental group were significantly increased (*p* < 0.05), and the expression level of *IL-6* was significantly decreased (*p* < 0.05).

## 4. Discussion

Inulin, as a natural prebiotic, has been extensively studied for its role in alleviating the stress response of weaned animals and enhancing their growth performance. da Silva, C.I. et al. [22] found that adding inulin to the feed of kids could increase the carcass rate of kids. Uerlings J et al. [23] reported that the average daily gain and average daily feed intake of weaned piglets supplemented with inulin increased significantly. de Campos, C.M. et al. [24] added 1.0% inulin to the diet of juvenile Piaractus mesopotamicus (Pacu) in intensive farming, which reduced the stress response of Pacu in high-density farming conditions, decreased serum cortisol concentrations, and simultaneously increased lysozyme concentration. The results of this experiment showed that there was no significant difference in the final weight of kids across all groups. However, the addition of inulin helped to reduce the F/G of kids and improved their growth performance.

Chemical stress regulators, such as COR, exert immunosuppressive effects, thereby increasing susceptibility to infectious diseases and potentially affecting the bioavailability of nutrients [25]. This study showed that inulin effectively lowered COR concentrations and mitigated stress in kids. Weaning stress significantly contributes to oxidative stress in young livestock within the context of animal farming. Research has indicated that, in intensive farming systems, the mortality rate of weaned kids can reach 40% due to the inadequate activities of antioxidant enzymes in breast milk [26]. Consequently, enhancing the activity of intestinal antioxidant enzymes is crucial for alleviating oxidative stress. Furthermore, inulin acts as an antioxidant, with the capacity to scavenge reactive oxygen species [27]. An experimental study in mice demonstrated that inulin supplementation effectively increased the activities of GSH-Px and SOD in the colon and reduced the concentrations of MDA [28]. In this study, supplementation with inulin enhanced the activity of antioxidant enzymes in the serum and intestines of kids and significantly decreased MDA concentration. A decrease in MDA concentrations in young livestock, therefore, mitigates cellular and tissue damage [29]. It can be concluded that supplementing weaned kids with an appropriate amount of inulin can effectively increase the activity of antioxidant enzymes, enhance their antioxidant capacity, and alleviate oxidative stress induced by weaning.

The main mechanisms of action of inulin include reducing stress-related indicators in young livestock, improving intestinal morphology, and thereby enhancing the absorption efficiency of nutrients [30]. VH, CD, and VCR are important indicators for evaluating the absorptive capacity of the small intestine, and they are closely related to digestive function [31]. Yuan et al. [32] found that adding inulin tended to decrease the ileal VCR in weaned calves, while their growth performance was improved. Consistent with previous studies, the results of this study demonstrated that inulin significantly reduced the VCR in the small intestine of weaned kids. This further confirms that inulin exerts a positive effect on improving intestinal morphology and function. In the intestine, oxidative stress can disrupt the function of intestinal tight junctions, increase intestinal permeability, facilitate the entry of pathogenic microorganisms into the intestine, and thereby trigger intestinal and systemic diseases [33]. Jiang et al. [34] showed that serum concentrations of D-LA and DAO can directly reflect the integrity of the intestinal physical barrier and the extent of intestinal stress-induced damage, while excessive LPS can damage the intestinal mucosal barrier and increase intestinal permeability [35]. In this study, the serum concentrations of D-LA, DAO, and LPS in all inulin-treated groups were significantly lower than those in the control group, which indicates that inulin can effectively maintain the integrity of the intestinal barrier structure and alleviate intestinal damage caused by weaning stress in kids. Inulin’s mechanism of action involves inhibiting the growth of Gram-negative bacteria through its prebiotic effect, thereby reducing oxidative stress and inflammatory responses induced by LPS [36]. However, in the 0.5% inulin group, the effect was not optimal. It may be that excessive intake of inulin can disrupt the homeostasis of the intestinal flora, leading to intestinal disturbances [37].

VFAs play a crucial role in maintaining the chemical barrier of the intestinal tract. They are produced by intestinal microorganisms via anaerobic fermentation of dietary fiber and can effectively lower the pH of the intestinal lumen, inhibit the growth of pathogenic microorganisms, and enhance the absorption efficiency of nutrients [38]. Zhu et al. [39] confirmed that inulin can increase the concentration of VFAs in the intestine. Acetic acid can enhance the ability of bifidobacteria to inhibit pathogenic bacteria and reduce inflammatory responses [40]. Butyric acid provides energy for the small intestinal epithelial cells, increases mucin production, and promotes the enhancement of intestinal tight junction barrier function [41]. Butyric acid and propionic acid can also regulate intestinal inflammation by inducing the differentiation of regulatory T cells [42]. This study found that 0.3% and 0.5% inulin significantly increased the concentrations of acetic acid, propionic acid, and TVFA in the cecum. Although the increase in butyric acid concentration was not significant, a gradual upward trend in butyrate concentration was observed with rising inulin dosage. This discrepancy from previous research findings may be associated with animal species and inulin dosage. In the 0.1% inulin group, the effect was not significant, possibly because low-dose inulin provides limited fermentation substrates, which were insufficient to fully activate the metabolic activity of the acid-producing bacterial community.

The weaning process disrupts the intestinal morphology of young livestock, leading to a reduction in digestive enzyme secretion [43] and impairing the absorptive capacity of the small intestine in young livestock. Intestinal digestive enzymes are mainly secreted by the digestive glands, and their activity directly affects the digestive and absorptive capacity of the digestive system for nutrients. They are also key indicators reflecting feed intake [44]. The results of this study showed that the 0.3% and 0.5% inulin groups had significantly increased activities of α-amylase, lactase, trypsin, chymotrypsin, and lipase in the jejunum, with the 0.3% inulin group exhibiting the best effect. This indicates that inulin could alleviate weaning stress in kids by enhancing the intestinal barrier and increasing digestive enzyme activities.

Serum biochemical indices are important markers for evaluating the overall health status and immune function of animals. Among these, indices such as TG, TC, UN, and AKP play significant roles in animal health and immune function. Zhao et al. [45] found that adding inulin to the diet of animals can effectively reduce the concentrations of TC and TG in serum. Consistent with the findings of this study, inulin supplementation significantly decreased the concentrations of TG and TC, which indicates that inulin can regulate lipid metabolism to improve the health status of animals [46]. Idowu, M. et al. [47] found that the content of UN in cattle serum was negatively correlated with their immune capacity. In addition, serum AKP plays an important role in bone formation [48]. In this experiment, kids fed with inulin supplementation had lower serum UN concentrations and higher serum AKP levels. By regulating the levels of UN and AKP in serum, inulin can enhance immune capacity and accelerate bone development in young livestock, thus improving their growth performance.

Inulin can stimulate the secretion of immunoglobulins (such as IgA and IgM) by relevant immune cells (including B lymphocytes and dendritic cells), inhibit the expression of pro-inflammatory factors (e.g., IL-6, IFN-γ, and TNF-α), and enhance the immune response by activating the complement system [49]. Meanwhile, IL-1β, IL-6, and TNF-α produced by keratinocytes are key to activating innate immunity by stimulating dendritic cells and T cells. IL-6 and TNF-α are produced by IL-1β through a molecular cascade and thus exhibit the same trend of change [50]. In this experiment, compared with the control group, the concentrations of serum IL-6 and TNF-α in inulin-supplemented groups showed the same downward trend. In addition, sIgA is widely present on the surface of intestinal mucosa, where it can recognize and coat pathogenic bacteria to prevent their invasion [51]. A relevant study has shown that inulin can be directly recognized by surface receptors of intestinal immune cells; increase the concentrations of sIgA, IgA, and IgG in the intestine; and induce immune regulation [52]. In this study, inulin supplementation significantly increased the concentrations of immunoglobulins in each segment of the small intestine, thereby enhancing the immune response of kids to alleviate stress.

Tight junction proteins are a key component of the intestinal physical barrier, and their expression levels reflect the integrity of the intestinal barrier [53]. Inulin can upregulate the expression of genes related to intestinal epithelial cells, strengthen their tight junctions, and maintain the integrity of the intestinal barrier [54]. This study showed that the 0.3% and 0.5% inulin groups had significantly increased gene expression levels of *ZO-1*, *CLDN-3*, and *OCLN* in the jejunum. This indicates that an appropriate amount of inulin can maintain the integrity of the intestinal physical barrier by enhancing intestinal tight junctions and reducing intestinal permeability. TGF-β1, a member of the transforming growth factor-β superfamily, effectively inhibits the secretion and activity of other inflammatory factors [55]. Yasuda et al. [56] found that dietary supplementation of inulin in weaned piglets reduced the expression of the inflammatory factors *TNF-α* and *SLC11A1* in the colon. This study also found that the expression levels of *TGF-β1* and *IL-10* in the jejunum were upregulated, while the expression level of *IL-6* was downregulated in the 0.3% and 0.5% inulin groups.

After LPS binds to macrophages and T cells, the cells synthesize *IL-6* [57], indicating that *IL-6* levels are positively correlated with inflammatory responses. In contrast, the primary function of IL-10 is associated with terminating inflammatory responses, helping to eliminate infectious organisms and reduce damage to host tissues [58]. In this experiment, inulin supplementation resulted in a reduction in *IL-6* expression and an increase in *IL-10* expression in the jejunum, indicating that inulin helps mitigate inflammatory responses in the jejunum of kids. This study, by integrating findings on serum and intestinal immune factors, confirmed that inulin can increase immunoglobulin concentrations and alleviate intestinal barrier damage induced by stress. Future research could focus on the gut microbiota–immune–brain axis regulated by inulin supplementation.

## 5. Conclusions

The results in this study suggest that supplementation with 0.3% inulin yields optimal effects, significantly enhancing immune function and antioxidant capacity in kids. In contrast, 0.5% inulin disrupts the homeostasis of the intestinal flora, interfering with nutrient absorption. An appropriate amount of inulin can not only enhance the immunity and antioxidant capacity of kids but also increase the levels of intestinal tight junction proteins and reduce intestinal permeability. These findings provide theoretical support for the application of inulin as a feed additive to improve the intestinal health of livestock.

## Figures and Tables

**Table 1:** The composition and nutrient concentration parameters of the dietary formula (DM basis).

Items	Content
**Ingredients (%)**	
Pennisetum	50.00
Corn	29.00
Soybean meal	10.00
Bran	7.50
Premix ^1^	2.00
CaHPO_4_	0.50
NaHCO_3_	0.50
NaCl	0.50
Total	100.00
**Nutrient levels**	
ME/(MJ/kg) ^2^	10.81
Crude protein	17.20
Crude fat	2.90
Crude ash	6.30
NDF	30.33
ADF	16.58
Ca	0.97
*p*	0.53

^1^ The premix provided the following per kg of diets: Vitamin A 17500 IU, Vitamin D3 6200 IU, Vitamin E 750 mg, Vitamin B3 500 mg, Vitamin H 1.5 mg, Cu 0.2 g, Fe 1 g, Mn 0.8 g, Zn 1 g, I 10 mg, Se 10 mg, and Co 7.5 mg. ME, metabolic energy; ADF, acid detergent fiber; NDF, neutral detergent fiber. ^2^ ME was calculated in accordance with NY/T 816-2021.

**Table 2:** Primer information.

Gene Name	Primer Sequence (5’-3’)	GenBank Accession No.	Product Length (bp)
*TJP1(zo-1)*	F1: TTCCTAAGACAGCAGGGGGA	XM_018066116.1	121
	R1: AACTTGGTCTGGACTGGCTG		
*CLDN-3*	F1: CACCATCATCCGGGACTTCT	XM_018041071.1	179
	R1: GCGCCGAGTAGACGATCTTG		
*OCLN*	F1: CTGCTGCCGACGAGTACAAT	XM_018065680.1	135
	R1: TCCGTCGGTCGTAATCTCCA		
*TGFB-1*	F1: ACAATTCCTGGCGCTACCTC	NM_001314142.1	199
	R1: CCGGAACTGAACCCGTTGAT		
*IL-6*	F1: CTTCACAAGCGCCTTCAGTC	NM_001285640.1	126
	R1: AGTAGTCTGCTTGGGGTGGT		
*IL-10*	F1: CATGGGCCTGACATCAAGGA	XM_005690416.3	113
	R1: GCCTTGCTCTTGTTTTCGCA		
*GAPDH*	F1: ACGTGTCCGTTGTGGATCTG	XM_005680968.3	142
	R1: AAGTCGCAGGAGACAACCTG		

**Table 3:** Comparison of effects of inulin on growth performance of weaned kids.

Items	Control	AM HCl	0.1% Inulin	0.3% Inulin	0.5% Inulin	SEM ^1^	*p*-Value
Days 1 to 25							
Initial BW (kg)	9.03	9.00	9.00	9.02	8.97	0.21	1.000
Final BW (kg)	10.37	10.70	10.41	10.63	10.53	0.14	0.831
ADG (g/d)	53.60	68.00	56.43	64.33	62.5	5.15	0.704
ADFI (g/d)	517.66 ^b^	501.10 ^b^	537.75 ^b^	553.45 ^a^	527.50 ^b^	5.78	0.039
F/G	9.66 ^a^	7.37 ^c^	9.52 ^a^	8.60 ^b^	8.44 ^b^	0.14	<0.001
Days 26 to 40							
Final BW (kg)	11.55	12.07	11.62	12.05	11.83	0.13	0.521
ADG (g/d)	78.67 ^c^	91.11 ^a^	80.68 ^c^	94.67 ^a^	86.94 ^b^	7.26	0.038
ADFI (g/d)	587.13	548.34	588.28	592.89	561.01	4.23	0.097
F/G	7.46 ^a^	6.01 ^d^	6.83 ^b^	6.26 ^c^	6.45 ^c^	0.28	<0.001
Days 1 to 40							
ADG (g/d)	63.06 ^c^	76.77 ^a^	65.55 ^b^	75.73 ^a^	71.67 ^ab^	4.87	0.022
ADFI (g/d)	552.41 ^a^	524.74 ^b^	563.02 ^a^	560.21 ^a^	544.26 ^ab^	6.44	0.002
F/G	8.76 ^a^	6.83 ^c^	8.58 ^a^	7.40 ^b^	7.59 ^b^	0.16	<0.001

^1^ SEM, standard error of the mean. BW: body weight, ADG: average daily gain, ADFI: average daily feed intake, F/G: feed-to-gain ratio. Control: kids were fed with basal diet; AM HCl: kids were fed supplemented with 0.05‰ aureomycin HCl; 0.1% inulin, 0.3% inulin, and 0.5% inulin groups: kids were fed with 0.1%, 0.3%, and 0.5% of inulin, which were added respectively to the basal diet. ^a–c^ Within a row, different letters indicate significant differences (*p* < 0.05).

**Table 4:** Effects of inulin on intestinal morphology indexes of weaned kids.

Items	Control	AM HCl	0.1% Inulin	0.3% Inulin	0.5% Inulin	SEM ^1^	*p*-Value
Duodenum							
VH (μm)	523.93 ^d^	736.50 ^b^	846.53 ^a^	888.80 ^a^	656.40 ^c^	35.35	<0.001
CD (μm)	393.83	368.23	434.60	395.93	347.37	14.04	0.394
VCR	1.37 ^b^	2.00 ^a^	2.00 ^a^	2.26 ^a^	1.90 ^a^	0.10	0.022
Jejunum							
VH (μm)	706.43 ^b^	698.13 ^b^	693.53 ^b^	808.33 ^a^	885.70 ^a^	22.61	0.001
CD (μm)	393.07 ^b^	351.70 ^b^	374.50 ^b^	400.13 ^b^	465.83 ^a^	11.80	0.004
VCR	1.80	1.99	1.85	2.04	1.90	0.04	0.290
Ileum							
VH (μm)	365.03 ^c^	425.93 ^b^	428.87 ^b^	480.60 ^a^	476.70 ^a^	11.41	<0.001
CD (μm)	320.57 ^c^	232.00 ^d^	332.20 ^c^	320.40 ^c^	372.60 ^a^	13.08	<0.001
VCR	1.14 ^c^	1.84 ^a^	1.29 ^c^	1.50 ^b^	1.28 ^c^	0.07	<0.001

^1^ SEM, standard error of the mean. VH: villus height, CD: crypt depth, VCR: villus–crypt ratio. Control: kids were fed with basal diet; AM HCl: kids were fed supplemented with 0.05‰ aureomycin HCl; 0.1% inulin, 0.3% inulin, and 0.5% inulin groups: kids were fed with 0.1%, 0.3%, and 0.5% of inulin, which were added respectively to the basal diet. ^a–d^ Within a row, different letters indicate significant differences (*p* < 0.05).

## Data Availability

The data that support the findings of this study are available from the corresponding author upon reasonable request.
